# Complete Genome Sequences of Three Lactobacillus gasseri Urine Isolates Obtained from Postmenopausal Women

**DOI:** 10.1128/mra.00481-22

**Published:** 2022-08-08

**Authors:** Brianna M. Shipman, Amber Nguyen, Belle M. Sharon, Philippe E. Zimmern, Nicole J. De Nisco

**Affiliations:** a Department of Biological Sciences, The University of Texas at Dallas, Richardson, Texas, USA; b Department of Urology, The University of Texas Southwestern Medical Center, Dallas, Texas, USA; Loyola University Chicago

## Abstract

Lactobacillus gasseri frequently colonizes the lower urinary tract of healthy women. However, the role of L. gasseri in urinary tract health and the genes required for urinary tract colonization are poorly understood. Herein, we announce the complete genome sequences of three Lactobacillus gasseri isolates collected from the urine of postmenopausal women.

## ANNOUNCEMENT

Lactobacillus gasseri is a commensal bacterium that is resistant to low pH and bile salts and can colonize the human gut, vagina, and urinary tract (1). While its role in the urinary microbiome (urobiome) is comparatively poorly understood, L. gasseri is considered protective in the vaginal and gut microbiomes, where it controls the chemical niche through production of antimicrobial compounds, including bacteriocins (i.e., gassericin), lactic acid, and hydrogen peroxide (1). However, while L. gasseri is largely seen as a commensal organism, it has recently been found to occur more often in the urobiome of women with urgency urinary incontinence, indicating that much is still unknown about its role in the urobiome (2).

Before this work, only nine complete L. gasseri genomes had been deposited in the NCBI database, of which none were urine isolates. The dissemination of complete genome assemblies for urinary isolates of L. gasseri will improve our understanding of genetic adaptations to the urinary niche, including extrachromosomal elements like conjugative plasmids. As such, we are excited to report the complete genomic assemblies for three L. gasseri isolates collected from the urine of postmenopausal women as part of institutional review board-approved studies (STU 032016-006 and MR 17-120).

“Clean-catch” midstream urine was collected from three women, two without clinical urinary tract infection (UTI) history and one with UTI history but no UTI at the time of collection. Urine was plated onto De Man, Rogosa, and Sharpe (MRS) agar. The plates were incubated microaerophilically at 35°C using the GasPak EZ Campy pouch system (BD) for 3 days. Species were identified using Sanger sequencing of the PCR-amplified 16S rRNA gene (8F and 1492R primers) as described previously (3) and MegaBLAST (BLAST v2.10.0) (4). After identification, isolated colonies were cultured in MRS broth under the same conditions. Then, the DNeasy blood and tissue kit (Qiagen) was used to extract genomic DNA, which was then analyzed using the 260/280-nm absorbance ratio as well as with agarose gel electrophoresis.

For the long reads, Oxford Nanopore libraries were created using the ligation sequencing kit (SQK-LSK109) and barcode expansion kit 1-12 (EXP-NBD104) before being sequenced using a MinION device with R9 FLO-MIN106 flow cells. The long reads were analyzed using ONT MinKNOW for live base calling, barcode trimming, and demultiplexing and were then quality trimmed using NanoFilt v2.6.0 (5). The trimmed reads were checked for quality using NanoStat v1.2.0, with reads of <200 bp and Phred scores of <7 being discarded. Illumina library prep was performed using the Nextera DNA Flex library prep kit, after which 2 × 150-bp paired-end reads were generated using a NextSeq 500 instrument. Read quality checking and trimming was performed using CLC Genomics Workbench v12.0.3, discarding all reads with a Phred score below 20 and a length of below 15 bp.

Closed hybrid assemblies were created using the normal mode of Unicycler v0.4.8 (6), SPAdes v3.13.0 (7), Racon v1.4.10 (8), and Pilon v1.2.3 (9, 10). The closed genomes were then rotated to the start of either the *dnaA* or *repA* gene, when present. The genomes were analyzed for quality using QUAST v5.0.2 (11) and checked for completeness using the bacteria ortholog set on the gVolante v1.2 (12) server with Bandage v0.8.1 (13) and BUSCO v1 (14). All genomes were complete. Finally, the number of coding sequences and GC content were determined using Geneious Prime v2020.0.5, and the genomes were annotated using the NCBI Prokaryotic Genome Annotation Pipeline v4.11 (15, 16). For all analyses, default parameters were utilized.

### Data availability.

The genome sequences are available at GenBank (BioProject accession number PRJNA761982). The BioSample and SRA accession numbers may be found in Table 1.

**TABLE 1:** Accession numbers, assembly parameters, and isolate characteristics of three urinary Lactobacillus gasseri strains obtained from postmenopausal women with different UTI histories

Strain	Host health state	BioSample accession no.	SRA accession no^*a*^	No. of raw reads	No. of trimmed reads	ONT read *N*_50_ (bp)	Read depth (×)	GenBank accession no.	Type of contig (circular)	Total length (bp)	GC content (%)	No. of CDSs^*b*^
Lg637_C84	Never UTI	SAMN23313746	SRX13999865 (I), SRX13998806 (O)	8,108,286 (I), 258,821 (O)	8,019,100 (I), 258,365 (O)	6,123	590 (I), 443 (O)	CP087763	Chromosome	1,860,447	35.0	1,717
								CP087764	Plasmid	81,131	32.4	87
Lg1199_C173	Never UTI	SAMN23313747	SRX13999866 (I), SRX13998807 (O)	7,865,502 (I), 595,961 (O)	7,777,799 (I), 594,839 (O)	3,426	532 (I), 613 (O)	CP087959	Chromosome	1,928,547	34.8	1,781
								CP087960	Plasmid	74,843	32.7	73
								CP087961	Plasmid	54,525	34.9	66
								CP087962	Plasmid	23,715	33.4	34
Lg1266_C155	History of rUTI, no current UTI	SAMN23313748	SRX13999867 (I), SRX13998808 (O)	7,354,438 (I), 588,684 (O)	7,272,480 (I), 587,615 (O)	3,758	569 (I), 714 (O)	CP087761	Chromosome	1,776,837	35.1	1,628
								CP087762	Plasmid	43,072	35.9	51

a O, ONT; I, Illumina.

b CDSs, coding sequences.
